# A *CsWRKY48* Gene from Tea Plants Intercropped with Chinese Chestnut Plays an Important Role in Resistance to Biotic and Abiotic Stresses

**DOI:** 10.3390/ijms252413526

**Published:** 2024-12-17

**Authors:** Jianzhao Wang, Yikai Gong, Meng Li, Yan Bai, Tian Wu

**Affiliations:** College of Landscape Architecture and Horticulture, Southwest Forestry University, Kunming 650224, China; wangjzswfu@swfu.edu.cn (J.W.); gongyikai@swfu.edu.cn (Y.G.); limeng@swfu.edu.cn (M.L.); baiyan@swfu.edu.cn (Y.B.)

**Keywords:** tea plants intercropped with Chinese chestnut, *CsWRKY48*, trichomes, cold resistance, insect resistance

## Abstract

Tea plant (*Camellia sinensis*) is an important horticultural crop. The quality and productivity of tea plants is always threatened by various adverse environmental factors. Numerous studies have shown that intercropping tea plants with other plants can greatly improve the quality of their products. The intercropping system of Chinese chestnut (*Castanea mollissima*) and tea plants is an agricultural planting model in which the two species are grown on the same piece of land following a specific spacing and cultivation method. Based on a comparative transcriptome analysis between Chinese chestnut tea intercropped plantations and a pure tea plantation, it was found that the expression levels of the *WRKY* genes were significantly upregulated under the intercropping pattern. In this study, we cloned a candidate gene, *CsWRKY48*, and verified its functions in tobacco (*Nicotiana tabacum*) via heterologous transformation. The contents of protective enzyme activities and osmoregulatory substances were significantly increased, and the trichomes length and density were improved in the transgenic tobacco lines. This phenotype offered an enhanced resistance to both low temperatures and aphids for transgenic lines overexpressing *CsWRKY48*. Further analysis indicated that the *CsWRKY48* transcription factor might interact with other regulators, such as CBF, ERF, MYC, and MYB, to enhance the resistance of tea plants to biotic and abiotic stresses. These findings not only confirm the elevated resistance of tea plants under intercropping, but also indicate a potential regulatory network mediated by the WRKY transcription factor.

## 1. Introduction

Tea plants are a well-known cash crop in the world. Tea plants are not only threatened by biotic factors such as bacteria, fungi, and pests, but also abiotic threats such as salinity, low temperatures, drought, and high temperatures during their growth [1]. When tea plants are subjected to biotic and abiotic stresses, the normal growth of the tea plants is inhibited, resulting in their death in severe cases. However, tea plants have evolved mechanisms to respond to these stresses. For example, the level of expression of relevant resistance genes in tea plants will change, the synthesis of antioxidant substances will be accelerated, the stability of the cell wall will be enhanced, and other adaptive changes to minimize the damage caused by the stress on the tea plants will occur [2,3,4]. When the threat is diminished, these response mechanisms can return to the state before the stress and continue to maintain the various life activities of the plant [5]. Transcription factors (TFs) play an important role in these response mechanisms.

*WRKYs* play key roles in several signaling pathways and regulatory networks critical to plant defense responses. Plants have developed complex molecular mechanisms to reduce the harmful effects of cold temperatures and insect attacks, which involve processes that control the expression of stress-response-related genes [6]. They bind specifically to W-box (T) TGACC (A/T) sequences in the promoter regions of target genes, regulating their expression [7,8,9]. For instance, *OsWRKY76* significantly increased cold resistance in rice, and this cold resistance decreased when *OsWRKY76* was knocked out [10]. When *Acer truncatum* was exposed to low-temperature stress, all *AtWRKY* transcription factors were upregulated [11]. Similarly, *VvWRKY28* in grape improved low-temperature stress tolerance in *Arabidopsis thaliana* through heterologous genetic transformation [12]. Genome-wide association analyses and forward genetics studies identified *WRKY* as a factor that increases resistance to sugarcane aphid (*Melanaphis sacchari*) in *Sorghum bicolor* [13]. In chrysanthemum, *CmWRKY42* expression was significantly upregulated under salinity, low temperatures, and aphid stress [14]. These studies demonstrate the crucial role of *WRKY* TFs in plant resistance to both biotic and abiotic stresses.

Intercropping could make the best use of land resources and increase yields and income per unit area. It could make more effective use of natural resources such as light energy, water, and nutrients, and improve the efficiency of resource use. Different crops have different resource requirements in the growing process, and intercropping could lead to a more reasonable distribution and utilization of these resources, thus reducing the waste of resources [15]. By planting multiple crops, intercropping could reduce the economic risks brought about by single-crop disasters or market fluctuations. If one crop suffered a reduction in production or loss of sales due to weather, pests, diseases, or changes in market demand, the other crops might still be able to maintain good yields and marketability, thus stabilizing economic income [16,17]. Chinese chestnut tea intercropped tea plantations are the most typical tea plant intercropping mode. With a tea plant light saturation point of 30,000–50,000 LX, compared with pure tea plantations, Chinese chestnut tea intercropped tea plantations could significantly improve the photosynthetic capacity of tea plantations to prevent the occurrence of the ‘midday depression of photosynthesis’ phenomenon. Chinese chestnut tea intercropped tea plantations could also provide tea plantations with the appropriate shade, so the ecological environment of the tea plantation is more conducive to growth. The appropriate temperature for the growth of tea plants is 20–30 °C, and when the ambient temperature is higher than 30 °C, the growth rate of tea plants will be significantly slowed down. Tea plants intercropped with Chinese chestnut can control the appropriate temperature for the growth of tea plants between 20 and 30 °C. In the spring, due to shade provided by Chinese chestnut plants, the wind speed in the tea plantation will be reduced, reducing the heat exchange. In summer, the shading of Chinese chestnut plants effectively reduces the net radiation of Chinese chestnut tea intercropped tea plantations, preventing the adverse effects of high temperatures on tea plants. In winter, the cover of fallen Chinese chestnut leaves will effectively reduce the heat exchange between surface long-wave radiation and soil air, which is beneficial for soil insulation [18]. In this study, we identified a gene whose expression was significantly induced, *CsWRKY48*, from the transcriptomic data of tea plants intercropped with Chinese chestnut vs. pure tea plantations [19]. We analyzed the expression patterns of *CsWRKY48*. To further understand the function of *CsWRKY48*, we took low-temperature (4 °C) and aphids stresses as examples to explore the role of *CsWRKY48* in the regulation of tea plant resistance to biological and abiotic stresses. This research will not only enrich the understanding of the molecular regulation mechanism of the WRKY gene family but also provide useful information for further exploring the function and regulatory mechanism of the *CsWRKY48* gene under biological and abiotic stresses in *Camellia sinensis*.

## 2. Results

### 2.1. qPCR Analysis of WRKY48 Transcription Factors

**Figure 1:**
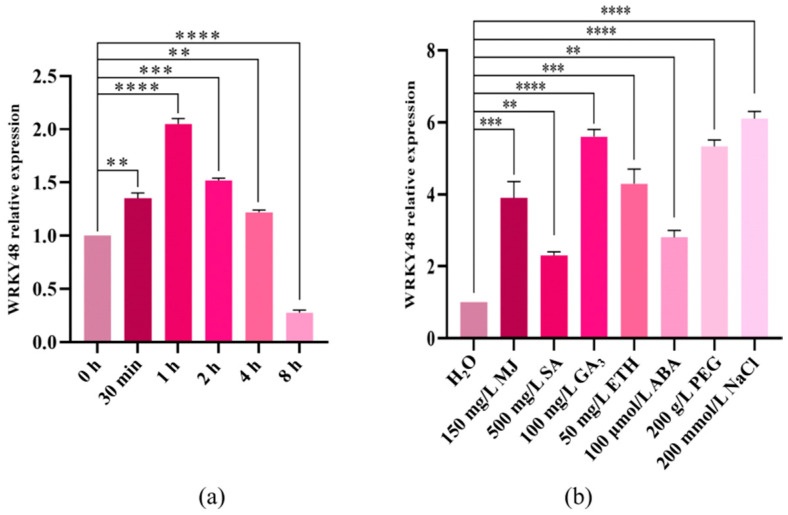
Expression analysis of *WRKY48*. (**a**) *WRKY48* expression under low temperature; (**b**) *WRKY48* expression under H_2_O (as a control), MJ (Methyl Jasmonate), SA (Salicylic acid), GA3 (Gibberellin A3), ETH (Ethylene), ABA (Abscisic acid), PEG (Polyethylene glycol), and NaCl. Note: The bar chart represents the mean of three biological replicates, with error bars showing standard deviations. Asterisks indicate statistical significance based on one-way analysis of variance (** *p* < 0.01, *** *p* < 0.001, and **** *p* < 0.0001).

The expression of *WRKY48* showed an initial increase followed by a decrease when subjected to low-temperature treatment. *WRKY48* reached its peak expression at 1 h of low-temperature treatment, which was 2.32 folds higher than at 0 h. These data suggest that *WRKY48* responds to low-temperature stress (Figure 1a).

After treatment with various hormones, PEG, and NaCl, the expression of *WRKY48* was significantly higher compared to water treatment. The highest expression levels of *WRKY48* were observed following the GA_3_, PEG, and NaCl treatments, showing 5.74, 5.68, and 6.13 folds greater expression than the water treatment (Figure 1b).

### 2.2. Gene Cloning and Sequence Analysis

**Figure 2:**
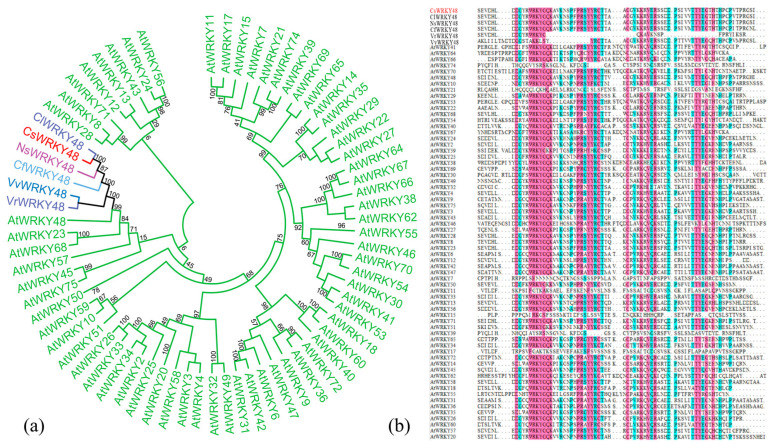
Analysis results of amino acid sequences for CsWRKY48. (**a**) Phylogenetic tree and (**b**) sequence analysis. Note: The target protein was marked with a red, ClWRKY48 (*Camellia lanceoleosa*, KAI8010938.1), NsWRKY48 (*Nyssa sinensis*, KAA8531200.1), CfWRKY48 (*Cornus florida*, XP059632820.1), VvWRKY48 (*Vitis vinifera*, RVW69378.1), and VrWRKY48 (*Vitis rotundifolia*, KAJ9699830.1).

*WRKY48* was cloned from tea plants intercropped with Chinese chestnut. Multiple sequence alignment and phylogenetic analysis revealed a high similarity between this gene and WRKY TFs in *Theaceae*, leading to its designation as CsWRKY48 (Figure 2a). *CsWRKY48* spanned 960 bp and contained an open reading frame encoding 317 amino acids. Sequence comparison of CsWRKY48 with Arabidopsis WRKY proteins revealed that CsWRKY48 contained a typical WRKYGQK domain (Figure 2b).

### 2.3. Genetic Transformation in Tobacco and Screening of Transgenic Tobacco Lines

Tobacco leaves were infiltrated with *Agrobacterium tumefaciens* containing the *pCAMBIA1300-CsWRKY48* overexpression vector, which induced resistant buds on MS3 media with hygromycin. These buds, upon reaching 1–2 cm in size, were transferred to MS4 media for seedling strengthening. Once the seedlings grew to 3–5 cm, they were moved to MS5 media for rooting and further plant development. qRT-PCR analysis revealed a transgenic efficiency of 95%.

**Figure 3:**
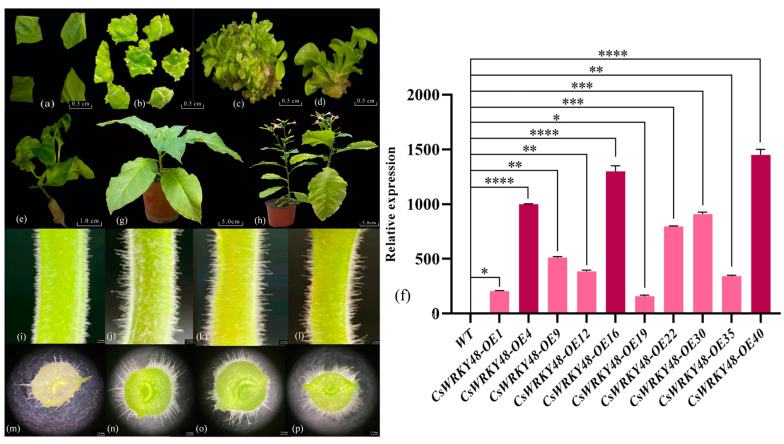
Genetic transformation, positive seedling screening, and phenotypic observation of transgenic tobacco. (**a**) Tobacco leaf discs infected with *Agrobacterium tumefaciens*; (**b**) induction of resistant buds; (**c**) expansion of resistant buds; (**d**) isolation of resistant buds; (**e**) formation of independent lines; (**f**) expression analysis of transgenic lines; (**g**) transgenic tobacco after transplanting; (**h**) seeds harvested from mature transgenic tobacco for subsequent experiments; (**i**,**m**) control tobacco plants; (**j**,**n**) *CsWRKY48*-OE4; (**k**,**o**) *CsWRKY48*-OE16; and (**l**,**p**) *CsWRKY48*-OE40. Note: The bar chart represents the mean of three biological replicates, with error bars showing standard deviations. Asterisks indicate statistical significance based on one-way analysis of variance (* *p* < 0.05, ** *p* < 0.01, *** *p* < 0.001, and **** *p* < 0.0001).

Twenty T1 tobacco lines were obtained, and their seeds were collected and sown to produce twenty T2 transgenic lines. DNA from the transgenic tobacco was used as a template for PCR, with primers designed for the hygromycin transferase gene. Ten positive lines were identified through PCR screening. Comparative analysis with the control tobacco plants revealed the following three high-expression lines: *CsWRKY48*-OE4, *CsWRKY48*-OE16, and *CsWRKY48*-OE40, with expression levels that were 997.67, 1350.74, and 1473.32 folds higher, respectively (Figure 3a–h).

### 2.4. Phenotype Observation of the CsWRKY48 Transgenic Lines

An analysis of the stem trichomes in the transgenic tobacco showed that they had more numerous and longer trichomes compared to the control plants (Figure 3i–l). Electron microscopy of freehand sections revealed that the control plants had approximately 10 trichomes per field of view (20×), with lengths ranging from 0.54 to 1.31 mm and an average length of 0.79 mm. In contrast, the transgenic plants exhibited 20–30 trichomes, with lengths ranging from 2.23 to 3.21 mm and an average length of about 2.72 mm. This resulted in trichome lengths and densities that were three folds higher than those of the control tobacco plants (Figure 3m–p). These increases in trichome length and density suggest an improved ability of transgenic tobacco to resist cold and sucking insects.

### 2.5. Phenotypic Analysis of Transgenic Tobacco After Low-Temperature Treatment and Aphid Feeding

**Figure 4:**
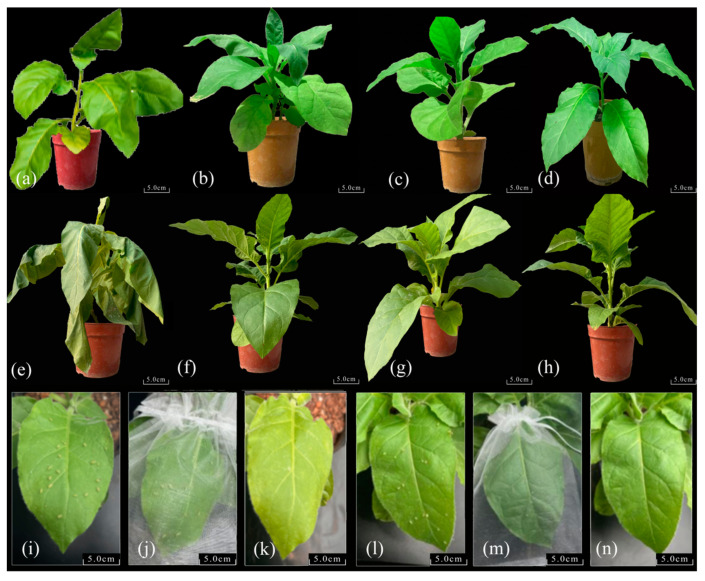
Phenotype analysis of transgenic tobacco after low-temperature treatment and aphid feeding. (**a**,**e**,**i**–**k**) WT (wild-type); (**b**,**f**) *CsWRKY48*-OE4; (**c**,**g**) *CsWRKY48*-OE16; (**d**,**h**) *CsWRKY48*-OE40; (**a**–**d**) after low-temperature treatment for 12h; (**e**–**h**) after low-temperature treatment for 24 h; (**i**–**n**) after aphid feeding for 12 h.

The average height of the transgenic and control tobacco plants was 42.35 cm, the leaf width was 11.27 cm, and the leaf length was 17.61 cm. No significant differences were observed in plant height, leaf length, or leaf width between the transgenic and control tobacco plants. However, the control tobacco plants showed slight wilting and yellowing after 12 h of exposure to low temperatures (Figure 4a), while the transgenic tobacco plants remained upright (Figure 4b–d). After 24 h of low-temperature treatment, the wilting and yellowing of the control tobacco plants increased further (Figure 4e), whereas the transgenic tobacco plants remained upright, except for a slight yellowing of the leaves (Figure 4f–h). There were sagging leaf margins and a pronounced yellowing of the control tobacco leaves after 12 h of aphid feeding (Figure 4i–k). However, there were no obvious differences in the transgenic tobacco after compared to before aphid feeding (Figure 4l–n).

### 2.6. Cold Resistance Analysis of CsWRKY48 Overexpressing Tobacco Plants

**Figure 5:**
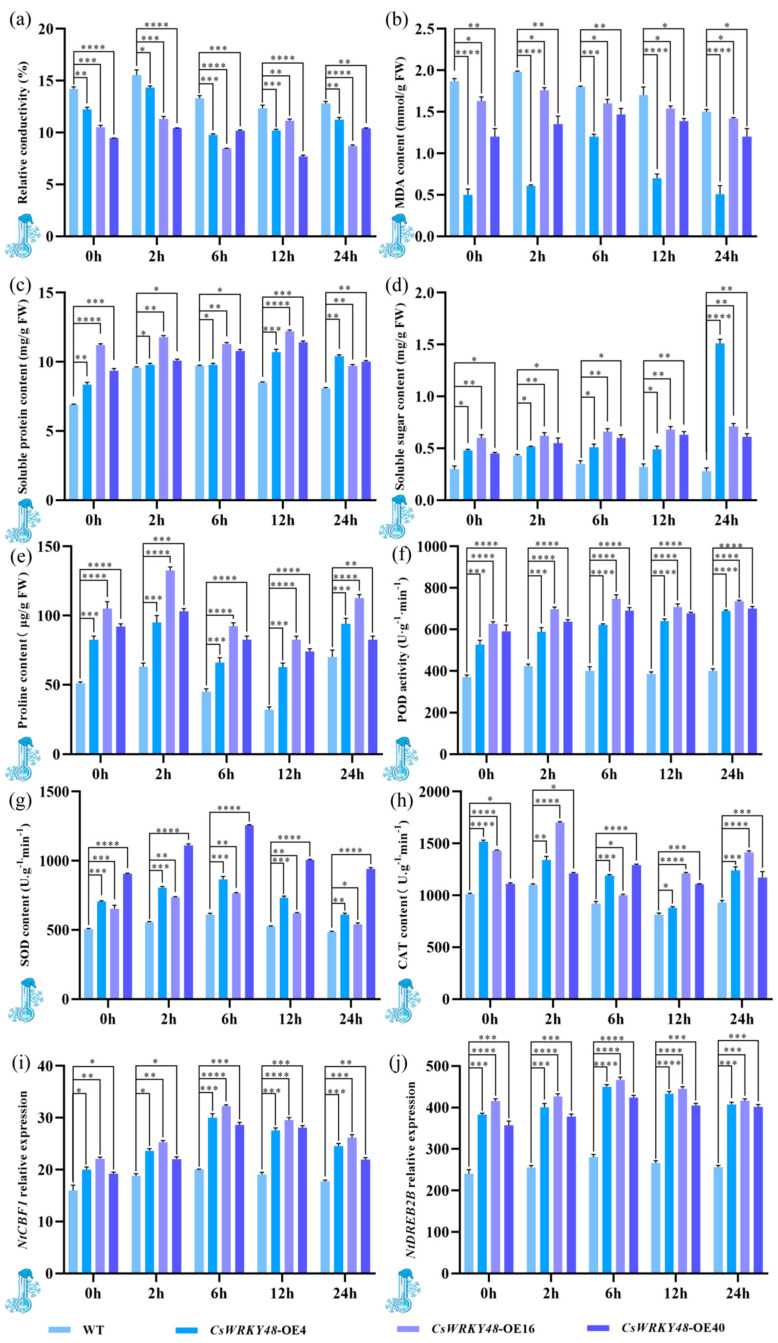
Physiological and biochemical indices and relative expression of two cold resistance genes in transgenic tobacco at 4 °C. (**a**) Relative conductivity; (**b**) MDA content; (**c**) soluble protein content; (**d**) soluble sugar content; (**e**) proline content; (**f**) POD activity; (**g**) SOD activity; (**h**) CAT activity; (**i**) relative expression of *NtCBF1*; and (**j**) relative expression of *NtDREB2B*. Note: The bar chart represents the mean of three biological replicates, with error bars showing standard deviations. Asterisks indicate statistical significance based on one-way analysis of variance (* *p* < 0.05, ** *p* < 0.01, *** *p* < 0.001, and **** *p* < 0.0001).

After treatment at 4 °C, the relative conductivity of the three transgenic tobacco lines were lower than before the treatment and significantly lower than that of the control plants. After 6 h of exposure to 4 °C, the relative conductivities of *CsWRKY48*-OE4 and *CsWRKY48*-OE16 reached their lowest values, which were 0.74 and 0.72 folds lower than before treatment, respectively. After 12 h, the relative conductivity of *CsWRKY48*-OE40 reached its lowest value, 0.72 folds lower than before treatment (Figure 5a). The malondialdehyde (MDA) content in the three transgenic lines was also lower than before treatment and significantly lower than that in the control tobacco plants. The MDA levels in the transgenic plants initially increased with treatment time and then decreased (Figure 5b).

The soluble protein content in the transgenic plants increased by an average of 1.87, 1.05, and 1.23 folds (Figure 5c); the soluble sugar content by 1.82, 1.71, and 1.78 folds (Figure 5d); and the free proline content by 1.04, 1.15, and 1.21 folds. After treatment at 4 °C, the levels of soluble protein, soluble sugar, and free proline in all three transgenic lines were higher than at 0 h and significantly higher than in the control tobacco plants, indicating a greater cold tolerance in the *CsWRKY48* transgenic tobacco compared to the control tobacco plant (Figure 5e).

Following 4 °C treatment, the transgenic lines also exhibited notable increases in the POD (Peroxidase), SOD (Superoxide Dismutase), and CAT (Catalase) activities. Specifically, the POD activity increased by 1.17, 1.14, and 1.01 folds (Figure 5f); the SOD activity by 1.15, 1.08, and 1.14 folds (Figure 5g); and the CAT activity by 1.13, 0.88, and 1.19 folds (Figure 5h). These enzyme activities followed the pattern of an initial increase followed by a decrease and were significantly higher than at 0 h and in the control tobacco plants, further suggesting an enhanced cold tolerance in the *CsWRKY48* transgenic lines.

Moreover, after 4 °C treatment, the relative expression of the cold resistance genes *NtCBF1* and *NtDREB2B* significantly increased compared to both 0 h and the control tobacco plants (Figure 5i,j). *CsWRKY48* significantly elevated the expression of cold-resistant genes, thereby improving the low-temperature tolerance of the plants.

### 2.7. Insect Resistance Analysis of CsWRKY48-Overexpressing Tobacco Plants

**Figure 6:**
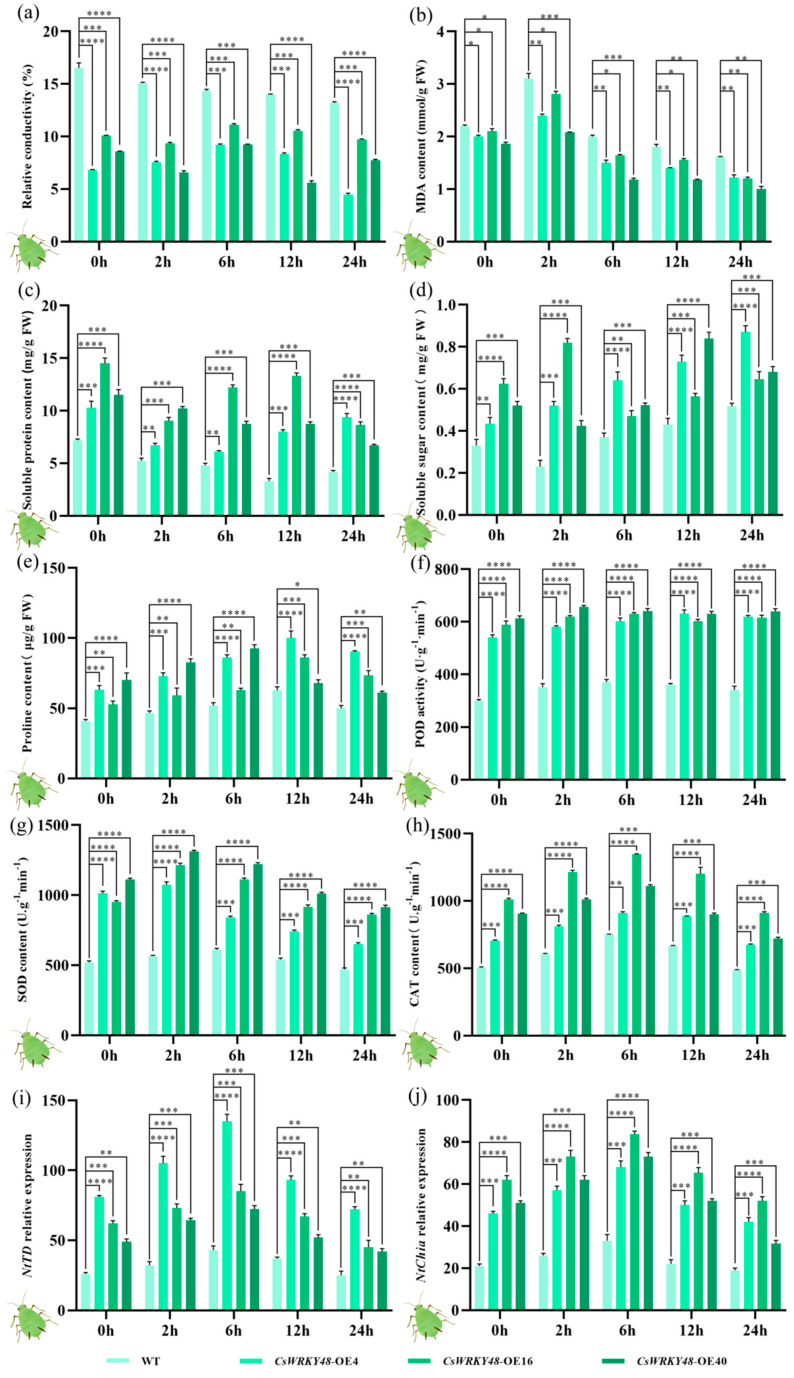
Antioxidant enzyme activities and the expression of two insect resistance genes in the control tobacco plant and the *CsWRKY48* transgenic plant after aphid feeding. (**a**) Relative conductivity; (**b**) MDA content; (**c**) soluble protein content; (**d**) soluble sugar content; (**e**) proline content; (**f**) POD activity; (**g**) SOD activity; (**h**) CAT activity; (**i**) relative expression of *NtTD*; and (**j**) relative expression of *NtChiA*. Note: The bar chart represents the mean of three biological replicates, with error bars indicating standard deviations. Asterisks denote statistical significance based on one-way analysis of variance (* *p* < 0.05, ** *p* < 0.01, *** *p* < 0.001, and **** *p* < 0.0001).

Following aphid feeding, the relative conductivity of the three transgenic tobacco lines decreased compared to 0 h and were significantly lower than that of the control tobacco plants. After 6 h of aphid feeding, the relative conductivities of *CsWRKY48*-OE4, *CsWRKY48*-OE16, and *CsWRKY48*-OE40 reached their peak values, which were 1.63, 1.12, and 1.16 folds higher than that before feeding, respectively (Figure 6a). The MDA content in the three transgenic lines was lower than that before feeding and significantly lower than that in the control tobacco plants. The MDA levels initially increased with treatment and then decreased; after 24 h of aphid feeding, the MDA content reached its lowest values, which were 0.65, 0.69, and 0.58 folds lower than that before feeding, respectively (Figure 6b).

The soluble protein content in the transgenic lines increased by 0.65, 0.76, and 0.77 folds on average (Figure 6c), while the soluble sugar content increased by 1.64, 1.02, and 1.26 folds (Figure 6d), and the free proline content by 1.18, 1.39, and 1.08 folds (Figure 6e). After aphid feeding, the levels of soluble proteins, soluble sugars, and free proline in all three transgenic tobacco lines were higher than before feeding and significantly higher than in the control plants, indicating a stronger insect tolerance in the *CsWRKY48* transgenic lines compared to the control tobacco plant.

The transgenic tobacco also showed notable increases in POD, SOD, and CAT activities following aphid feeding. These enzyme activities followed an increasing and then decreasing trend. The POD activity increased by 1.01, 1.05, and 1.02 folds on average (Figure 6f), the SOD activity by 0.81, 1.13, and 1.21 folds (Figure 6g), and the CAT activity by 1.02, 1.16, and 1.03 folds (Figure 6h). These activities were significantly higher than before feeding and in the control tobacco plants, indicating an enhanced insect tolerance in the *CsWRKY48* transgenic lines.

Moreover, after aphid feeding, the relative expression of two insect resistance genes, *NtTD* and *NtChia*, significantly increased compared to pre-treatment levels and the control tobacco plant (Figure 6i,j). This suggested that *CsWRKY48* promoted the expression of insect resistance genes, improving the plants’ ability to resist insect attacks.

### 2.8. CsWRKY48 TF Promoter Analysis

**Figure 7:**
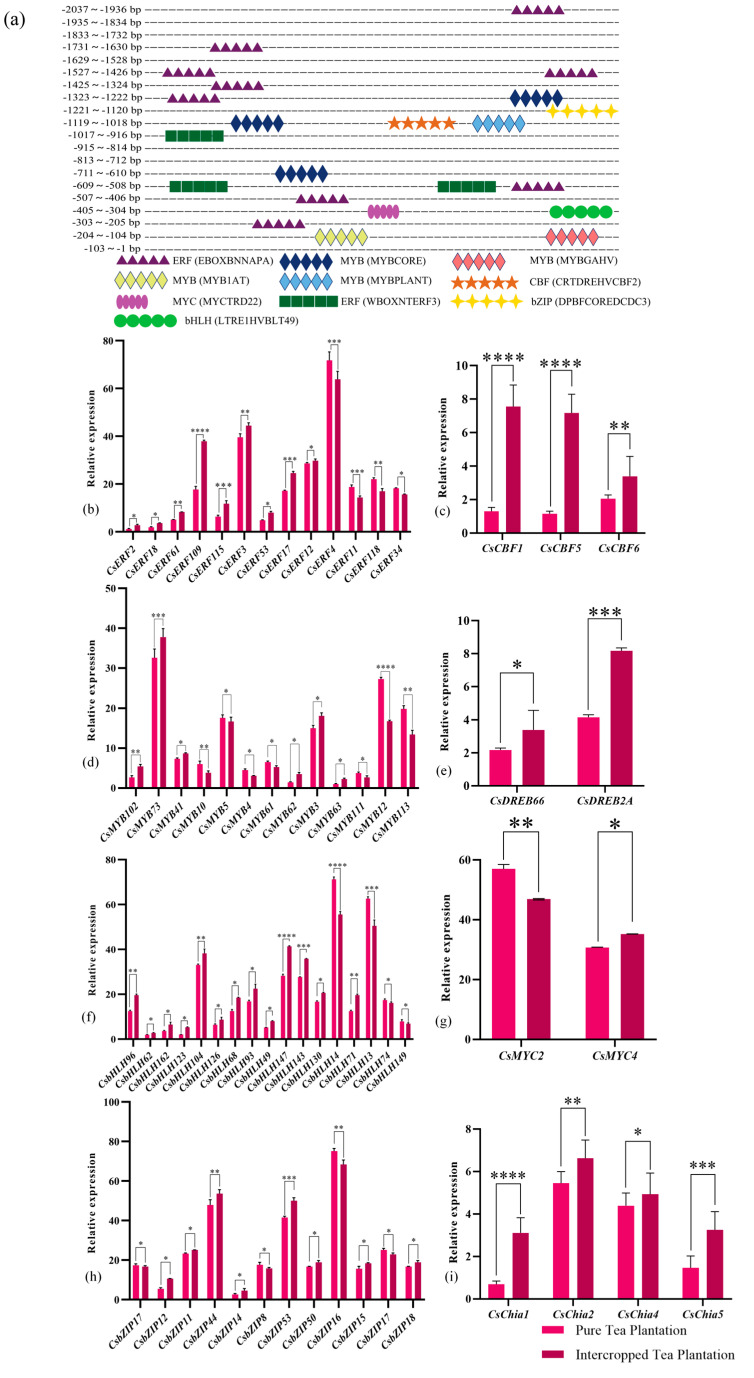
Prediction of cis-elements in the promoter of *CsWRKY48*, and analysis of the expression of relevant transcription factors and resistance genes in the transcriptomic data of the tea plants intercropped with Chinese chestnut. Note: (**a**) Prediction of cis-element in the promoter of *CsWRKY48*; analysis of the expression of relevant transcription factors and resistance genes in the transcriptomic data of the tea plants intercropped with Chinese chestnut. (**b**–**i**) Analysis of the expression of relevant transcription factors and resistance genes in the transcriptomic data of the tea plants intercropped with Chinese chestnut (* *p* < 0.05, ** *p* < 0.01, *** *p* < 0.001, and **** *p* < 0.0001).

The *CsWRKY48* promoter was cloned, and the full length of the promoter sequence was 2037 bp. The promoter sequence of *CsWRKY48* contained various core cis-elements, with notable action elements involved in the plant growth regulator response and stress resistance response. The plant growth regulator response elements included the ethylene response element EBOXBNNAPA; ABA response elements such as MYB1AT and MYBCORE; and the GA response element MYBGAHV. The resistance stress response elements included the flooding response element MYBCORE; the cold response elements CRTDREHVCBF2; and the drought response element MYCTRD22 (Figure 7a).

Similarly, the transcription factors capable of binding to these cis-elements were also found in the transcriptome data from the Chinese chestnut tea intercropped plantation. A total of 13 *ERF*s, 13 *MYB*s, 17 *bHLH*s, 12 *bZIP*s, 2 *MYC*s, 3 *CBF*s, and 2 *DREB*s were identified, among them, *CsERF109*, *CsMYB73*, *CsDREB2A*, *CsbHLH147*, *CsChia1*, *CsbZIP53*, and *CsMYC2* showed the most significant differences in expression, which were 2.24, 1.39, 1.91, 1.86, 2.54, 1.15, and 1.28 folds higher than those of the pure tea plantation; however, the expression levels of *CsERF4*, *CsCBF1*, *CsMYB12*, *CsbHLH14*, and *CsbZIP16* in the pure tea plantation were 1.12, 4.88, 1.58, 1.32, and 1.12 folds higher than those in the Chinese chestnut tea intercropped plantation (Figure 7b–h). In addition, there were significant changes in some functional genes from the transcriptomic data of tea plants intercropped with Chinese chestnut vs. pure tea plantations including *CsChia1/2/4/5* (Figure 7i) and *CsGSTU8/23/45* [19]. These differences in expression provide a clearer explanation for improving the resistance of tea plants in the Chinese chestnut tea intercropped plantations compared to those in pure tea plantations.

## 3. Materials and Methods

### 3.1. Plant Materials

Tender leaves (the second leaves from the top) from *C. sinensis ‘Yunkang No.10’* grown in Chinese chestnut tea intercropped plantations in Kunming were promptly frozen in liquid nitrogen and stored at −80 °C. Due to the lack of an efficient homologous transformation system in *C. sinensis*, tobacco was used as the model plant for heterologous genetic transformation.

### 3.2. Plant Growth Conditions

The Chinese chestnut tea intercropped tea plantations were located in the botanical garden of Southwest Forestry University, Kunming, Yunnan Province, with a soil pH of 5.0–5.5. Tobacco plants were cultivated in a tissue culture room maintained at a constant 25 °C, with a daily light exposure of 10–12 h and a humidity range of 65–75%.

A total of six different culture media were used in this study. YEB 1 (for *Agrobacterium tumefaciens* liquid medium), consisting of YEB + 100 mg/L Rif (Rifampin) + 100 mg/L Kan (Kanamycin) + 6.5 g/L Agar, and YEB 2, consisting of YEB + 100 mg/L Rif + 100 mg/L Kan, were used for spreading and shaking the bacteria during the transformation step. MS (Murashige and Skoog) medium was used at different stages, as follows: MS 1 (3% MS + 6.5 g/L Agar + 30 g/L sucrose) was employed for infection during genetic transformation. MS 2 (3% MS + 2.25 mg/L 6-BA (6-Benzylaminopurine) + 0.3 mg/L NAA (Naphthaleneacetic acid) + 6.5 g/L Agar) was applied in the genetic transformation process. MS 3 (3% MS + 2.25 mg/L 6-BA + 0.3 mg/L NAA + 20 mg/L Hyg (Hygromycin) + 400 mg/L Cef (Cephalosporin) + 6.5 g/L Agar) was used for the preliminary screening of transformants. Finally, MS 4 (3% MS + 0.1 mg/L 6-BA + 0.1 mg/L NAA + 20 mg/L Hyg + 400 mg/L Cef + 6.5 g/L Agar) was utilized for the rooting culture of transgenic tobacco.

### 3.3. Expression of WRKY48 Under Low Temperatures, Different Plant Growth Regulators, PEG, and NaCl

The differential expression of the *WRKY48* gene was analyzed from the transcriptome data of Chinese chestnut tea intercropped plantations and pure tea plantations. qPCR primers for the *WRKY48* and *Actin* genes were designed using NCBI Primer-BLAST and synthesized by Sangon Biotech (Shanghai, China). The relative expression levels of *WRKY48* were quantified using the 2^−ΔΔct^ method under the treatments of a low temperature (4 °C), different plant growth regulators, including MJ (methyl jasmonate), SA (methyl jasmonate), GA_3_ (gibberellin A3), ETH (ethylene), and ABA (abscisic acid), PEG (Polyethylene glycol), and NaCl. Cold resistance analysis was performed by exposing the tea plant leaves to 4 °C for different durations of 0 h, 30 min, 1 h, 2 h, 4 h, and 8 h.

### 3.4. The CsWRKY48 Gene Cloning

RNA was extracted following the protocol provided in the RNA extraction kit (Magen Biotech, Guangzhou, China). Specific cloning primers, P1-F and P1-R, were designed using the NCBI online platform. The cDNA, generated from reverse transcription, served as the template for amplifying the full-length sequence of *CsWRKY48*. The RT-PCR conditions were set as follows: 5 min at 94 °C (one cycle), 30 s at 94 °C (35 cycles), 30 s at 55 °C (35 cycles), 1 min at 72 °C (35 cycles), followed by 10 min at 72 °C (one cycle) and 10 min at 10 °C (one cycle) for a 50 μL reaction buffer. A gel recovery kit was used to purify the desired bands (Tiangen Biotech, Beijing, China). The target band was ligated into a cloning vector and transformed into *E. coli* receptor cells.

### 3.5. The CsWRKY48 Gene Promoter Cloning and Sequence Analysis

DNA was extracted from the tea plant leaves using the TIANGEN DNA Secure Plant Kit (Tiangen Biotech, Beijing, China), and the extracted DNA was evaluated via 1.0% agarose gel electrophoresis. Specific cloning primers, P11-F: GGCGTACCTCCAGCATCTAA and P11-R: GCTTGTAATTGGCTGACCTCC, were designed through NCBI’s online platform to amplify the target fragment using diluted DNA as the template in a PCR amplification system. The PCR products were analyzed by electrophoresis, and the desired bands were purified using a gel DNA recovery kit (TAKARA^TM^ Gel DNA Recovery Kit). The target band was then ligated into a cloning vector and transformed into *E. coli* receptor cells. Positive colonies were sequenced, and the obtained sequence, named *CsWRKY48pro*, was analyzed using New Place for promoter action elements.

### 3.6. Over Expression Vector Construction, Plant Transformation of CsWRKY48, and Identification of Transgenic Lines

The *BamHI* and *PstI* restriction enzymes were used to target specific genes, followed by linearization of the cloning vectors via agarose gel electrophoresis, gel cutting, and DNA recovery (TAKARA^TM^ Gel DNA Recovery Kit). The resulting cleaved product was purified using a DNA purification kit. The target fragment was then ligated into the pCAMBIA1300-35S vector and transformed into *E. coli* for positive clone screening and sequencing. The extracted plasmids were introduced into *Agrobacterium tumefaciens* GV3101.

Tobacco leaf discs (0.5 cm * 0.5 cm) were incubated for 10 min in Agrobacterium-infused MS1 media. After infection, the surface sap was removed from the leaves, which were placed dorsal side up on MS2 media for co-culturing in the dark. After 48 h on MS2 media, the leaves were transferred, dorsal side down, onto MS3 media to induce resistant buds and screen cultures. Once the resistant buds reached 1–2 cm in length, they were transferred to MS4 media. Upon reaching a consistent height of 3–5 cm, the plants were moved to MS5 media for rooting. DNA was extracted from the transgenic tobacco, and primers for hygromycin transferase and *CsWRKY48* genes were designed for the PCR screening of positive lines. All positive lines were further analyzed for gene expression via qPCR to identify those with higher expression levels. The empty vector pCAMBIA1300-35S was also transformed into tobacco plants as a control.

### 3.7. Observations on the Phenotype of Transgenic Tobacco

The length and density of tobacco trichomes were observed using the freehand slicing method and examined with an electron microscope (LEICA DM2500).

### 3.8. Low-Temperature Stress in Transgenic Tobacco

Six-week-old T2 generation control tobacco plants and transgenic tobacco seedlings with a uniform growth were subjected to 4 °C in a constant light incubator for 0, 2, 6, 12, and 24 h. Three biological replicates were performed for each line. After low-temperature treatment, the tobacco phenotypes were assessed, and the leaves were stored at −80 °C for further analysis.

### 3.9. Aphid Feeding Stress in Transgenic Tobacco

Six-week-old T2 generation control tobacco plants and transgenic tobacco seedlings with a uniform growth were selected. Twenty-four instar aphid larvae were placed on the second mature leaf from the top to feed for durations of 0, 2, 6, 12, and 24 h. Three biological replicates were conducted for each line. The tobacco phenotypes were observed, and combined samples from the 3rd and 4th leaves (from the top) were collected and stored at −80 °C for further analysis.

### 3.10. Determination of Physiological and Biochemical Indices of Transgenic Tobacco

The soluble protein content was measured using the Coomassie Brilliant Blue G-250 method, the soluble sugar content was determined via Anthrone Colorimetry, and the malondialdehyde content was assessed through Thiobarbituric Acid Colorimetry. The free proline content was measured using the acid ninhydrin method. The CAT (Catalase), POD (peroxidase), and SOD (superoxide dismutase) activities were detected using the UV absorption, Guaiacol, and Riboflavin NBT methods, respectively. Each parameter was tested three times, and the average values were recorded.

### 3.11. Expression Analysis of Stress-Related Genes in Transgenic Tobacco

qRT-PCR primers for the tobacco cold resistance genes *NtCBF1* and *NtDREB2B*, as well as the insect resistance genes *NtChiA* and *NtTD* (Table 1), were designed and synthesized by Sangon Biotech (Shanghai, China). RNA extracted from the tobacco leaves subjected to low-temperature and aphid feeding stress was quality checked, reverse-transcribed into cDNA, and then used for qRT-PCR. The expression levels of cold and insect resistance genes in the stress-treated control and transgenic tobacco plants were analyzed.

### 3.12. Statistical Analysis and Graphing of Data

The data were processed, analyzed, and visualized using IBM SPSS Statistics 22.0, GraphPad Prism 9, Adobe Illustrator 2023, and Photoshop 2023. Duncan’s multiple range test was employed to examine significant differences between groups, and one-way ANOVA was used for multiple comparisons. All data were presented as mean ± standard error of the mean. A *p* < 0.05 was considered to be statistically significant.

## 4. Discussion

### 4.1. Effects of Trichomes on Plant Stress Resistance

Trichomes play a crucial protective role in plants. This study demonstrated that transgenic tobacco exhibited both an increased trichome density and length compared to the control tobacco plants. These specialized epidermal structures were essential for plant defense against insects and pathogens, as well as for an enhanced resistance to freezing and other environmental stress. For example, in *Artemisia annua*, glandular secreting trichomes (GSTs) were regulated by specific *WRKY* TFs, such as *AaGSW2*, which influenced trichome germination. When *AaGSW2* was knocked out, trichome formation was affected [25]. Similarly, transcriptome data from tomato trichomes showed that transcription factors like *MYC*, *bHLH*, and *WRKY* could activate the *Solanum lycopersicum* terpene synthase promoter, promoting trichome growth [26,27,28]. The heterologous expression of *GhWRKY53* (*Gossypium hirsutum*) in *Arabidopsis thaliana* also significantly increased trichome density [29]. A trichome transcriptome database was established with a total of 27,195 alleles, including 743 annotated TFs. Among these, *MYC*, *bHLH*, and *WRKY* were shown to transiently transactivate *S. lycopersicum* terpene synthase promoters in *Nicotiana benthamiana* leaves, promoting trichome formation [30]. Previous studies have highlighted the importance of *MYC* and *bHLH* in trichome development, and their cis-elements were identified in the *CsWRKY48* promoter sequence. In the transcriptome data of tea plants from the Chinese chestnut tea intercropped plantations, the expression of *MYC* and *bHLH* were significantly higher than that of tea plants from the pure tea plantation. This suggested that *CsWRKY48* may have interacted with *MYC* and *bHLH* to co-regulate trichome growth, which should be verified in future studies. The role of trichomes as an important component of plant defense against insects and pathogens is obvious. However, whether this defense mechanism is specific or has a broad defense effect needs to be supported by more experimental data.

### 4.2. Regulation of CsWRKY48 Gene by Hormone Crosstalks

The *CsWRKY48* gene responded to various plant growth regulators. In this study, its expression significantly increased in transgenic tobacco following treatments with MJ, ABA, ETH, GA_3_, and SA. The promoter of *CsWRKY48* contained several cis-acting elements responsive to these hormones, including GA_3_-responsive elements (TAACAAAA-motif), JA-responsive elements (AACGTG-motif), and ABA-responsive elements (VAACCA-motif, ACACNNG-motif). This suggests crosstalk between the *CsWRKY48* gene and MJ, IAA, ETH, GA_3_, and SA pathways. Previous research supports the role of *WRKY* genes in hormone signaling and stress response. For example, *Eucalyptus grandis* (*EgWRKYs*) genes displayed diverse expression profiles under salt and cold stress, as well as hormone treatments (SA, JA, and BR), indicating their involvement in plant development and abiotic stress responses [31]. Eighty-four *IbWRKY* genes were identified in sweet potato (*Ipomoea batatas*) and classified into three major categories (I, II, and III), which showed different expression profiles under abiotic stresses (NaCl, PEG, heat, and cold) and hormonal treatments (ABA, ACC, JA, and SA) [32]. In *Caragana korshinskii*, *CkWRKY*s engage in crosstalk with ABA to enhance resistance to abiotic stress [33]. Similarly, in *Glycyrrhiza glabra*, *GgWRKY15* and *GgWRKY59* interacted with GA_3_ to enhance resistance to both biotic and abiotic stress [34]. In rice, SA interacted with *OsWRKY45* and promoted its expression to improve resistance to the white-backed planthopper [35]. Based on these findings, it is hypothesized that hormones such as SA, MJ, ABA, and GA_3_ play important roles in regulating plant resistance to biotic and abiotic stresses. When plants were subjected to adversity stress, enzymes and hormones in the plant were altered to affect a series of physiological activities or biochemical changes to produce adaptations to resist the stress. The interactions between transcription factors and phytohormones form complex signal networks that are linked by certain common components. In this study, we found that *WRKY48* responded to the induction of GA_3_, ETH, ABA, and other plant growth regulators, and we also found elements in the promoter region of *WRKY48* that responded to these plant growth regulators, and we think that these hormones might work together with *WRKY48* to form a regulatory network to increase the resistance of tea plants to biotic and abiotic stresses. However, the interactions between transcription factors and hormones were intricate, and the pathways through which they enhance plant resistance need to be further identified.

### 4.3. CsWRKY48 Enhancement of Cold and Insect Resistance in Plants via NtCBF1, NtDREB2B, NtChiA, NtTD, etc.

Cold and insect stress can significantly hinder plant growth and development. Identifying the genes involved in cold and insect resistance and understanding their molecular functions are essential for improving plant breeding for increased tolerance. Numerous studies have demonstrated that *WRKY* plays a crucial role in improving plant resistance and supporting plant growth and development. For example, in *Zea mays*, *ZmWRKY* significantly improved maize resistance to *Ostrinia furnacalis* [36]. In Arabidopsis, *WRKY*, *MYB*, *ERF*, *bHLH*, and *bZIP* were vital for stress-specific defense responses during aphid and *Pseudomonas syringae* attacks. In *Populus euramericana*, the overexpression of *PeWRKY31* increased salt tolerance and insect resistance in transgenic tobacco [37]. Additionally, the overexpression of *PmWRKY57* in Arabidopsis led to the upregulation of cold-responsive genes such as *AtCOR6.6*, *AtCOR47*, *AtKIN1*, and *AtRCI2A*, improving cold resistance [38]. In banana, the application of ABA induced the expression of *MaWRKYs* and increased endogenous ABA levels, which improved cold resistance [39]. Similarly, in rice, *OsWRKY76* interacted with *OsbHLH148* to transactivate the expression of *OsDREB2B*, thereby improving cold tolerance [10]. *CsWRKY48* directly binds to the W-box elements in the promoter of *CsGSTU8* and activates its expression, thereby increasing the drought tolerance of the tea plant [40]. *CsGSTU45* was differentially expressed in the Chinese chestnut tea intercropped transcriptome data, similar to drought tolerance, and we inferred that *CsWRKY48* regulated *CsGSTU45,* which should be verified in future studies. This study demonstrated that the expression of cold resistance genes (*NtCBF1* and *NtDREB2B*) and insect resistance genes (*NtChiA* and *NtTD*) was significantly upregulated in tobacco overexpressing the *CsWRKY48* gene. Tea plants might defend themselves against biotic or abiotic stresses through crosstalk in hormonal networks, as well as interactions between transcription factors. Combined with previous studies, we identified binding sites for transcription factors such as MYB, ERF, and MYC in the promoter of *CsWRKY48*. We supposed that *CsWRKY48* might be affected by endogenous plant growth regulators, as well as upstream transcription factors, to activate the expression of cold and insect resistance genes, such as *NtCBF1*, *NtDREB2B*, *NtChiA*, and *NtTD*, as a means of enhancing tea plant resilience.

## 5. Conclusions

**Figure 8:**
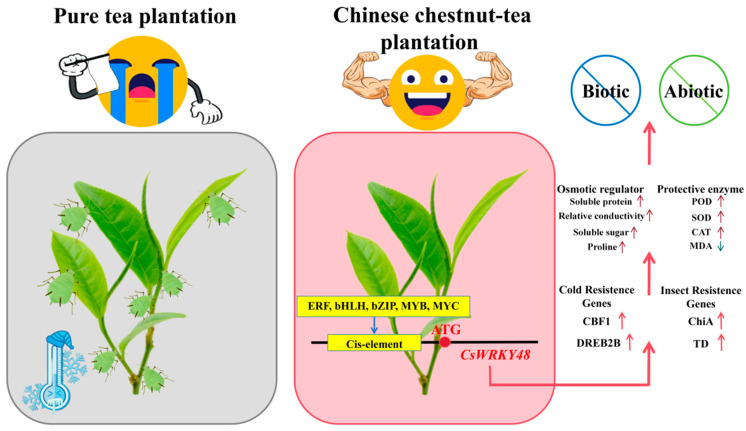
Resistance mechanism of Chinese chestnut tea plantation. Note: Grey box indicates that tea plants in pure tea plantations were susceptible to low temperatures and aphid stresses; pink box indicates that tea plants in Chinese chestnut tea intercropped tea plantations were more resistant to low temperatures and aphids. POD: Peroxidase; SOD: Superoxide dismutase; CAT: Catalase; ERF: Ethylene responsive factor; bHLH: Basic helix–loop–helix; bZIP: Basic leucine zippers; MYB: Myeloblastosis viral; CBF1: C-repeat binding factor; DREB2B: Dehydration responsive element binding; Chia: Chitinase; TD: Threonine deaminase.

In this study, we demonstrated that *CsWRKY48* significantly increased tea plants resistance to biotic and abiotic stresses in Chinese chestnut tea intercropped plantations. In this environment, changes in light exposure and allelopathic interactions may influence the hormonal levels in tea plants, triggering the activation of the *CsWRKY48* gene. This activation is mediated by the interaction of the *CsWRKY48* promoter with transcription factors such as *MYB*, *MYC*, *bHLH*, and *ERF* (Figure 8). *CsWRKY48* induced the upregulation of cold resistance genes like *CsCBF* and *CsDREB*, as well as insect resistance genes like *CsChiA*, which contributed to the overall plant health in Chinese chestnut tea intercropped plantation. Moreover, *CsWRKY48* affected trichome length and density, further improving resistance to cold and insects while increasing the fragrance of tea plants. Future research should focus on revealing how *CsWRKY48* interacts with plant growth regulators and transcription factors, such as *MYB*, *MYC*, *bHLH*, and *ERF*, to boost cold and insect resistance in tea plants. Understanding these interactions will be vital for uncovering the mechanisms that improve plant resilience in Chinese chestnut tea intercropped systems.

## Figures and Tables

**Table 1:** Primers synthesis of qPCR for cold resistance and insect resistance genes.

Primer Name	GenBank No.	Sequence
*NtEF1α-F*	NM001326165 [20]	TGGTTGTGACTTTTGGTCCCA
*NtEF1α-R*		ACAAACCCACGCTTGAGATCC
*NtCBF1-F*	NP001312156 [21]	GGATGAGGAGACGCTATTCTG
*NtCBF1-R*		TGTGAACACTGAGGTGGAGG
*NtDREB2B-F*	EU727156 [22]	CGGCCGCCCATCTGAGTC
*NtDREB2B-R*		AGGTGGAGGCAGCATTAGTC
*NtChiA-F*	P08252.2 [23]	GGCCTTGTGGAAGAGCCATA
*NtChiA-R*		CCAAATCCAGGGAGGCGATT
*NtTD-F*	AAG59585.1 [24]	ACATGGGTCAAGTTAGGCGG
*NtTD-R*		TATAGGGGTGGCAAATGGGC

## Data Availability

Data are contained within the article.
